# X Linkage of *AP3A*, a Homolog of the Y-Linked MADS-Box Gene *AP3Y* in *Silene latifolia* and *S. dioica*

**DOI:** 10.1371/journal.pone.0018972

**Published:** 2011-04-20

**Authors:** Rebecca H. Penny, Benjamin R. Montgomery, Lynda F. Delph

**Affiliations:** Department of Biology, Indiana University, Bloomington, Indiana, United States of America; Montreal Botanical Garden, Canada

## Abstract

**Background:**

The duplication of autosomal genes onto the Y chromosome may be an important element in the evolution of sexual dimorphism.A previous cytological study reported on a putative example of such a duplication event in a dioecious tribe of *Silene* (Caryophyllaceae): it was inferred that the Y-linked MADS-box gene *AP3Y* originated from a duplication of the reportedly autosomal ortholog*AP3A*. However, a recent study, also using cytological methods, indicated that *AP3A* is X-linked in *Silenelatifolia.*

**Methodology/Principal Findings:**

In this study, we hybridized *S. latifolia* and *S. dioica*to investigate whether the pattern of X linkage is consistent among distinct populations, occurs in both species, and is robust to genetic methods. We found inheritance patterns indicative of X linkage of *AP3A* in widely distributed populations of both species.

**Conclusions/Significance:**

X linkage of*AP3A* and Y linkage of *AP3Y*in both species indicates that the genes' ancestral progenitor resided on the autosomes that gave rise to the sex chromosomesand that neither gene has moved between chromosomes since species divergence.Consequently, our results do not support the contention that inter-chromosomal gene transfer occurred in the evolution of *SlAP3Y* from *SlAP3A*.

## Introduction

Sex-chromosome evolution in dioecious *Silene* has received considerable attention because the sex chromosomes are recently evolved, allowing exploration of early events in the process of sex chromosome evolution [1]. Chromosomal rearrangements can have important implications for sex-chromosome evolution [2]. Intragenomic translocations may limit recombination, although in *Silene* the role of chromosomal rearrangements in the cessation of X-Y recombination is yet uncertain [3].In the absence of recombination, genes linked to male traits could accumulate on the sex chromosomes, allowing differential expression between the sexes and facilitating the evolution of sexual dimorphism [4], [5]. Consequently it is of interest to understand the origin of Y-linked genes responsible for male traits. Such genes may have initially been located on autosomal ancestors of sex chromosomes, with male-specific roles evolving after Y-chromosome differentiation. In this case, for recently derived sex chromosomes, homologs of male-specific genes would be expected on the X chromosome. Alternatively, genes with male-specific function may have migrated to the Y chromosome after the chromosome's differentiation.In this case, such genes would lack a homolog on the X chromosome[6], [7].

A previous study using flow cytometry found evidence of autosomal inheritance of the *AP3A* gene in *Silene latifolia*, which is homologous to the Y-linked gene *AP3Y*,whichwould indicate that *AP3Y*'s location on the Y chromosome was attributable to a translocation event [8].Both *AP3A* and *AP3Y* contain MADS-box sequence motifs involved in floral development, with *AP3A* expressed in petals of both male and female flowers and *AP3Y* expressed in petals and stamens of male flowers [8], [9]. Many studies have cited Matsunaga et al. [8] as a unique example of gene duplication onto the *Silene* Y chromosome that has implications for understanding the evolutionary processes involved in the origination of heterogametic sex chromosomes [2], [10], [11].

We were motivated to investigate Xlinkage of the *AP3A* gene because of preliminary results from a previous study [12], in which we attempted to identify hybrids of *S. latifolia* and *S. dioica*following mixed-species pollen applications by the presence of an amplification product of the heterospecific sire's *AP3A* gene.We successfully amplified the *S. latifolia* specific product from males and females of *S. latifolia* but not *S. dioica*, and we were only able to successfully amplify the *S. latifolia* specific product from male but not female F1 hybrids[12].Similar results were found for the *S. dioica* specific *AP3A* product for crosses in the other direction, leading to the hypothesis that *AP3A* is X-linked in both species.

In this study, we used interspecific crosses between *Silene latifolia* and *S. dioica*,species that share a homologous Y chromosome, to further investigate X linkage of *AP3A* across multiple populations. The structure of the Y chromosome has been shown to vary within populations, which makes the study of multiple populations important for the generalization of possible translocation events [3]. It has also been suggested that segregation experimentsrepresent the most reliable approach to investigate sex-linkage of genes [13]. Given two cytological-isolation studies yielding conflicting results[8], [14], our segregation study presents a logical method to conclusively determine the genomic location of the focal gene. By crossing pure species *S. latifolia* and *S. dioica* individuals in both cross directions, X-linked genes from maternal species were isolated in male F1 individuals. The expectation is that if the *AP3A/X* gene (designated *SlAP3A* in *S. latifolia*and *SdAP3A* in *S. dioica*) is X-linked, the paternal species copy would be absent in male F1s and present in female F1s. Our results suggest that *AP3A* is X-linked in both species, indicating that the location of *AP3Y* on the Y chromosome is consistent with its ancestral condition and predates the divergence of *S. latifolia* and *S. dioica*.

## Results and Discussion

The presence of *SlAP3A* in all *S. latifolia* individuals, *SdAP3A* in all *S. dioica* individuals, and *AP3Y* in all male individuals of both species was confirmed as were the reciprocal absences of these genes in the alternate species/sex. In *S. dioica* x *S. latifolia* (dam x sire) crosses all male F1s (N = 9) amplified *AP3Y* and not *SlAP3A*, whereas all female F1s (N = 12) amplified *SlAP3A* (Table 1).Reciprocal crosses between the species showed a similar pattern: all male F1's (N = 15) amplified *AP3Y*and only one of fifteen males amplified a product determined to be *SdAP3A*. All female F1s in this cross direction (N = 19) amplified *SdAP3A* and not *AP3Y*. These results are consistent with X-linked, and not autosomal, inheritance of the *AP3A* gene in both *S. latifolia* and *S. dioica*.

As a result of high sequence similarity, some misamplifications of *SdAP3A* occurred in male F1s from *S. latifolia* x *S. dioica* crosses.Amplification with *SdAP3A* primers resulted in a product for two male F1s in each of crosses B, D, and E and one male F1 in cross D.These products, as well as the species-specific products from each parent, were sequenced and compared to determine the identity and origin of the F1 products. In all seven cases, the F1's sequence corresponded with the dam's *SlAP3A* sequence and differed from the sire's *SdAP3A* sequence at each of four sites with one base-pair substitution. This clearly suggests that the observed product in these F1 males resulted from misamplification of *SlAP3A*, and not the presence of *SdAP3A*.

Overall, out of 24 F1 males, only one (from cross C) showed a pattern of inheritance consistent with autosomal inheritance.The *SdAP3A* sequence of this male showed evidence of double peaks, indicative of amplification of two similar products. There were present two insertions (2bp and 4bp) identical to the *SdAP3A* sequence of the sire but not the dam. Furthermore, at the four polymorphic loci previously discussed, dominant peaks corresponded to the sire's sequence while lesser peaks were consistent with the dam's sequence. This outcome could be explained by aneuploidy, with inheritance of X and Y from the sire.

For further comparison, the sire from cross C was crossed to another *S. latifolia* female (Cross C^1^). One male F1 was present among the 8 seeds planted for this cross. Under relaxed PCR conditions for the amplification of *SdAP3A*, products were obtained from all 8 F1 individuals. Consistent with the hypothesis of X-linked inheritance, sequences of products from the 4 F1 females sequenced all corresponded with the sire's *SdAP3A* sequence, whereas the sequence of the F1 male corresponded to the dam's *SlAP3A* sequence. Thus, for this cross, 5 of 5 individuals showed patterns consistent with X linkage of *AP3A*.

The evidence that *AP3A* is located on the X chromosome in both *S. latifolia* and *S. dioica* suggests that the current location of this gene predates the divergence of these species. Additionally, the pattern of X-linked inheritance of *AP3A* was consistent among severalgeographically distinct populations. Another recent study similarly detected an X-linked homolog but no autosomal homolog for *AP3Y* for one accession of *S. latifolia*using laser microdissection[14]. Given our finding across multiple populations and matching results from a different seed source, it is unlikely that population-level differences are sufficient to account for the discrepancies in results between these findings and those of Matsunaga et al. [8]. Instead, impurities in flow cytometry may account for the detection of an *AP3* gene associated with autosomes in Matsunaga et al. [14]. While the presence of an X-linked homolog does not diminish the importance of *AP3Y* in understanding sex-specific evolution of sex-linked genes, it does suggest that autosomal gene duplication was not an element in its specialization.

## Methods

Five half-sib families were formed by crossing five *Silene dioica* males from geographically distinct populations in Skeppsvik Island, Sweden, Roscoff and Alençon, France, and Graubünden and Wallis, Switzerland (CrossesA-E) with the same *S. latifolia* female (from Virginia).Another *S. latifolia* female (from France) was crossed with the *S. dioica* sire from crossC to yield a sixth family (Cross C^1^).Four half-sib families were formed in the reciprocal direction from one *S. dioica* dam (from Roscoff, France) and four *S. latifolia*sires from populations in Virginia, Italy, Portugal and France (crosses F-I).Eight seeds from each cross were planted. Sets of individuals from these plantings were genotyped for each family until either results were obtained for at least two males and two females or all plants had been genotyped. Larger samples were obtained for several crosses, but due to mortality and skewed sex-ratios, only one female in cross D and one male in cross G were genotyped.Seeds were planted in sterilized potting soil and housed at the Indiana University greenhouses.

PCR amplification was performed using DNA extracted from young leaves (QiagenDNEasy kit).The same program was utilized for the amplification of *SlAP3A* and *SdAP3A* (95°C for 2.5 min, 56.7°C for 30 s, 72°C for 30 s (30 cycles); 72°C for 5 min).A different program was used for the amplification of *AP3Y* (95°C for 2 min; 94°C for 20 s, 58°C for 10 s (decrease by 2°C every other cycle), 65°C for 45 s (8 cycles); 94°C for 20 s, 50°C for 10 s, 65°C for 45 s (30 cycles); 65°C for 10 min).

The oligonucleotide primer sets used for PCR were as follows: 5′-AGAAGGTAAAGAACCTTGAAG-3′ and 5′-ATACTGGAGATAACACAGCCT-3′ for *SlAP3A*, 5′-TGCAAGAGCAGAGAAAGT-3′ and 5′-GGTCGCAAACCAGTAGTTTAT-3′ for *SdAP3A*, 5′-AGATTAGTCGAAGGATAG-3′ and 5′-ATATTCGAGACAACATTG-3′ for *AP3Y*.Using the above primers, agarose gel electrophoresis yielded single fragment products for each amplification.The lengths of the fragments for *SlAP3A*, *SdAP3A*, and *AP3Y* were approximately 400 bp, 900 bp, and 700 bp, respectively. The identity of PCR products from parental individuals was verified through a nucleotide BLAST search (NCBI) of *AP3A* sequences from each dam used and the seven paternal individuals that we sequenced.

The presence of *AP3Y* in F1 individuals was used to determine the sex of these plants, and these results were later verified by flowering observations.Two male and two female plants from each full-sib family were then amplified for the putatively autosomal gene specific to the paternal species (*SlAP3A* or *SdAP3A*).

PCR products were verified by sequencing for maternal individuals used in crosses A-I (not cross C^1^), for seven of nine paternal individuals, and for 16 out of 35 F1 individuals in the *S. latifolia* x *S. dioica* crosses (Indiana Molecular Biology Institute, Applied Biosystems 3730 automated sequencing system, Applied BiosystemsBigDye Terminator ver3.1).Sequencing was also completed in instances where PCR products were suspected to be misamplifications of the homologous sequence from the maternal genome.Consensus sequences were assembled, viewed and edited using CodonCode Aligner (Version 3.5.7, CodonCode Corporation) and were compared to parental sequences for both genes using CLUSTAL X (version 2.0.10).

**Table 1:** Status of *AP3Y* and *AP3A* genes in hybrids of *Silene latifolia* and *S. dioica*.

**A. ** ***Silene latifolia*** ** dam by ** ***S. dioica*** ** sires**							
	**A**	**B**	**C**	**D**	**E**	**C^1^**	**Total**
**Female offspring**							
*AP3Y*	0	0	0	0	0	0	**0**
*SdAP3A*	4	2	2	1	3	7	**19**
**Male offspring**							
*AP3Y*	2	2	2	4	4	1	**15**
*SdAP3A*	0	0	1	0	0	0	**1**
**B. ** ***Silene dioica*** ** dam by ** ***S. latifolia*** ** sires**							
	**F**	**G**	**H**	**I**	**-**	**-**	**Total**
**Female offspring**							
* AP3Y*	0	0	0	0	-	-	**0**
* SlAP3A*	2	5	3	2	-	-	**12**
**Male offspring**							
* AP3Y*	4	1	2	2	-	-	**9**
* SlAP3A*	0	0	0	0	-	-	**0**

Status was determined through presence or absence of amplification product and, in some cases, comparison of product sequences with sequences of known identity. Letters represent separate half-sib families. ^1^Different dam but same sire as cross C.
